# *LAMP2* as a Biomarker Related to Prognosis and Immune Infiltration in Esophageal Cancer and Other Cancers: A Comprehensive Pan-Cancer Analysis

**DOI:** 10.3389/fonc.2022.884448

**Published:** 2022-04-21

**Authors:** Shan-peng Liu, Xiao-min Li, Dan-man Liu, Shu-huan Xie, Shao-bo Zhang, Yu Li, Ze-feng Xie

**Affiliations:** ^1^ Thoracic Surgery Department, The First Affiliated Hospital of Shantou University Medical College, Shantou, China; ^2^ Breast Surgery Clinics, Guangdong Province Women and Children Hospital, Guangzhou, China

**Keywords:** *LAMP2*, esophageal cancer, pan-cancer, diagnostic, prognosis, immune infiltration

## Abstract

Esophageal cancer (ESCA) is a common malignant tumor with poor prognosis. Accumulating evidence indicates an important role of lysosomal-associated membrane protein 2 (LAMP2) in the progression and development of various cancers. In this study, we obtained RNA-sequencing raw count data and the corresponding clinical information for ESCA samples from The Cancer Genome Atlas and Gene Expression Omnibus databases. We comprehensively investigated the expression and prognostic significance of *LAMP2* and relationships between *LAMP2* expression and prognosis, different clinicopathological parameters, and immune cell infiltration in ESCA. We also obtained the differentially expressed genes between the high *LAMP2* expression and low *LAMP2* expression groups in ESCA and performed a functional enrichment analysis of the 250 linked genes most positively related to *LAMP2* expression. Moreover, we performed the pan-cancer analysis of *LAMP2* to further analyze the role of *LAMP2* in 25 commonly occurring types of human cancer. We also verified and compared the expression of *LAMP2* in 40 samples of human ESCA tissue and adjacent tissues. The results indicated that *LAMP2* expression was significantly upregulated in ESCA and various human cancers. In addition, *LAMP2* expression was associated with certain clinicopathological parameters, prognosis, and immune infiltration in ESCA and the other types of cancer. Our study represents a comprehensive pan-cancer analysis of *LAMP2* and supports the potential use of the modulation of *LAMP2* in the management of ESCA and various cancers.

## Introduction

Esophageal cancer (ESCA), which includes esophageal squamous cell carcinoma (ESCC) and esophageal adenocarcinoma (EAC), is a leading cause of cancer-related mortality worldwide (1). Currently, the diagnosis and prognosis of ESCA depend on various factors, including the clinicopathological stage, histological type, tumor size, age, and treatment sensitivity. Early clinical symptoms in patients with ESCA are usually insidious and mild (2, 3). Most patients have a locally advanced or metastatic disease at the time of diagnosis. Despite recent advances in the treatment of ESCA, including molecular-marker-based diagnosis, radiomics, targeted therapies, and immunotherapy, long-term survival rates remain relatively low (4–6). Therefore, it will be of great clinical value to understand the molecular mechanisms underlying the occurrence and progression of ESCA in order to explore effective and novel biomarkers and improve the diagnosis and prognosis of patients with ESCA.

The metabolic function of lysosomes is extremely important and increasingly recognized. The role of lysosomes was not novel in the degradation of cellular machinery (3). Previous research has shown that the functional status and spatial distribution of lysosomes are associated with the proliferation, energy metabolism, invasion and metastasis, immune escape, drug resistance, and tumor-associated angiogenesis of cancer cells (7–9). The *LAMP2* gene encodes lysosomal-associated membrane protein 2 (LAMP2), which is a single transmembrane protein located at the limiting membrane of lysosomes and late nuclear endosomes. *LAMP2* has three isoforms, *LAMP2a*, *2b*, and *2c*, which are involved in autophagy (10). Accumulating evidence indicates that lysosomes play an important part in various cancers; this has raised interest in the role of *LAMP2* in cancer progression. For example, the absence of glycolytic metabolism and vascularization produces an acidic microenvironment in the early stages of *in situ* breast cancer, leading to an increase in *LAMP2* on the plasma membrane and tumor progression (11). In addition, *LAMP2* expression on the plasma membrane promotes the adhesion of cancer cells to the extracellular matrix, basement membrane, and endothelium, as well as the migration potential of cancer cells during metastasis (12). *LAMP2* is also highly expressed in poorly differentiated human colorectal cancer, prostate cancer, hepatocellular carcinoma, adenoid cystic carcinoma, and lung adenocarcinoma and represents a novel molecular biomarker for these cancer types (13–17). Therefore, whether *LAMP2* could also be a pan-cancer molecular biomarker, especially in ESCA, is worth further study.

In this study, the expression of *LAMP2* in ESCA was investigated using The Cancer Genome Atlas (TCGA) (18) and Gene Expression Omnibus (GEO) (19) databases. Subsequently, we explored the correlations between *LAMP2* expression and various clinicopathological features and between *LAMP2* expression and the prognostic value in different clinicopathological features. The differentially expressed genes (DEGs) between high *LAMP2* expression and low *LAMP2* expression groups in ESCA were obtained. We investigated the association between the expression of the top 50 related genes and *LAMP2* expression, followed by a functional enrichment analysis of the top 250 linked genes most positively related to *LAMP2* using Gene Ontology (GO) terms and Kyoto Encyclopedia of Genes and Genomes (KEGG) pathways. We also analyzed the relationships between immune infiltration parameters and different expression levels of *LAMP2* in ESCA. To further analyze whether *LAMP2* followed the same expression rules in other tumors, *LAMP2* expression in 25 types of human common cancer was obtained from TCGA. Moreover, we analyzed the relationships between *LAMP2* expression and prognostic value, different immune infiltration parameters, and different clinicopathological features in other human cancers. Our results may help to find novel immunotherapy treatments for patients with ESCA and other cancers, as well as provide new ideas and directions for clinical research on pan-cancer therapy.

## Materials and Methods

### Data Collection

The RNA-sequencing (RNA-seq) data and relevant clinical data across 33 tumor types and normal tissues of 15,776 samples were downloaded from The Cancer Genome Atlas (http://cancergenome.nih.gov) and the Genotype-Tissue Expression (GTEx) database (https://www.gtexportal.org/home/-index.html), which contained the extraction of ESCA: GTEx normal (n=653); TCGA paraneoplastic (n=13); and TCGA tumor (n=182). Furthermore, the GSE23400 (n=208) (20), GSE33426 (n=71) (21), GSE53625 (n=358) (22), and GSE45670 (n=38) (23) datasets were used for the validation of the expression difference analysis. The samples with missing expression data were excluded from the study. In addition, the downloaded data were used to explore the relationship of *LAMP2* with various clinicopathological parameters (including the pathologic stage, N stage, M stage, and residual tumor), diagnosis [receiver operating characteristic (ROC)], prognosis [overall survival (OS), progression-free interval (PFI), and disease-specific survival (DSS)], and gene coexpression and bioenrichment [GO, KEGG, and gene set enrichment analysis (GSEA)] in ESCA (ESCC and EAC).

The following tumor types were included: bladder urothelial carcinoma (BLCA); breast-invasive carcinoma (BRCA); cervical squamous cell carcinoma and endocervical adenocarcinoma (CESC); cholangiocarcinoma (CHOL); colon adenocarcinoma (COAD); rectum adenocarcinoma (READ); lymphoid neoplasm diffuse large B-cell lymphoma (DLBC); glioblastoma multiforme (GBM); glioma (GBMLGG); head and neck squamous cell carcinoma (HNSC); kidney chromophobe (KICH); kidney renal clear cell carcinoma (KIRC); kidney renal papillary cell carcinoma (KIRP); acute myeloid leukemia (LAML); brain lower-grade glioma (LGG); liver hepatocellular carcinoma (LIHC); lung adenocarcinoma (LUAD); lung squamous cell carcinoma (LUSC); mesothelioma (MESO); ovarian serous cystadenocarcinoma (OV); pancreatic adenocarcinoma (PAAD); prostate adenocarcinoma (PRAD); rectal adenocarcinoma (READ); sarcoma (SARC); skin cutaneous melanoma (SKCM); stomach adenocarcinoma (STAD); testicular germ cell tumors (TGCTs); thyroid carcinoma (THCA); thymoma (THYM); uterine corpus endometrial carcinoma (UCEC); and oral squamous cell carcinoma (OSCC).

### Tumor Immune Estimation Resource 2.0

Tumor Immune Estimation Resource 2.0 (TIMER 2.0; http://timer.cistrome.org/) is an updated interactive web server that allows the investigation and visualization of tumor immunity. TIMER 2.0 assists in finding the associations between gene expression, mutations, immune infiltration, and survival characteristics in the TCGA cohort (24). In this study, the expression of *LAMP2* in multiple cancer types was assessed using the “Exploration-Gene_DE” model. The “Immune-Gene” module was employed to explore the relationship between *LAMP2* expression and the infiltration levels of immune cells (neutrophils, macrophages, dendritic cells, B cells, CD8+ T cells, and CD4+ T cells) on a pan-cancer basis.

### Gene Expression Profiling Interactive Analysis 2

Gene Expression Profiling Interactive Analysis 2 (GEPIA2; http://gepia2.cancer-pku.cn/#index) is a free web portal for the differential gene expression analysis of TCGA and GTEx data (25). In the present study, *LAMP2* expression was analyzed using the TCGA-ESCA dataset. *LAMP2* expression in ESCA and paraneoplastic tissue samples was studied using the “Expression DIY” module in GEPIA2.

### UALCAN

The UALCAN database (http://ualcan.path.uab.edu/index.html) can be used to analyze online data (TCGA, The MET500 metastatic cancer cohort, and Clinical Proteomic Tumor Analysis Consortium) and clinical data with respect to the differential gene expression between tumor and normal tissues (26). UALCAN was used here to study *LAMP2* expression and its pan-cancer relationships with protein expression in the following 10 subtypes: k1 overexpression of proteasome complex proteins, glycolysis proteins, and pentose phosphate pathway proteins), k2 (adaptive immune system related; associated with T-cell activation; expression of major histocompatibility complex proteins), k3 (innate immune system related; overexpression of complement system proteins; involvement of eosinophils, neutrophils, mast cells, and macrophages; hypoxia signature), k4 (represents basal-like breast cancer; overexpression of YAP1 and MYC targets), k5 (epithelial signature; normoxia signature; overexpression of YAP1 and MYC targets; overexpression of oxidative phosphorylation and the tricarboxylic acid (TCA) cycle proteins), k6 (stromal related; overexpression of matrix metallopeptidases; Wnt and Notch pathway signatures; hypoxia signature), k7 (stromal related; overexpression of collagen VI proteins; Wnt and Notch pathway signatures), k8 (overexpression of Golgi apparatus-related proteins; Ras pathway signature), k9 (found in KIRC cases only; overexpression of hemoglobin complex proteins), and k10 (overexpression of endoplasmic reticulum (ER)-related proteins and steroid biosynthesis pathway proteins).

### PrognoScan

PrognoScan (http://dna00.bio.kyutech.ac.jp/PrognoScan/index.html) is a database for the meta-analysis of the prognostic value of genes (27). It was used here to validate the use of *LAMP2* in prognosis in cancers using the GEO dataset.

### TISIDB

TISIDB (http://cis.hku.hk/TISIDB/) is a website for exploring tumor-immune system interactions that integrates numerous data types (28). In this study, TISIDB was used to construct a heat map showing the correlations among immunomodulators, lymphocytes, chemokines (or receptors), and gene expression. In addition, TISIDB was used to explain the correlations of *LAMP2* expression with immune subtypes and molecular subtypes of tumors.

### Tumor Immune Dysfunction and Exclusion

The tumor immune dysfunction and exclusion (TIDE) algorithm (http://tide.dfci.harvard.edu/query/) provides data to support the studies of T-cell dysfunction and immunotherapy resistance in cancer based on large clinical datasets (29). In this study, the general predictive ability of *LAMP2* with respect to treatment response in different cancer types was compared with those of nine standardized biomarkers for tumor immune response using the “Biomarker Evaluation” model, including TIDE, the microsatellite instability (MSI) score, tumor mutational burden (TMB), cluster of differentiation 274 (CD274), cluster of differentiation 8 (CD8), interferon-γ (IFNG), T-cell clonality (T.Clonality), B-cell clonality (B.Clonality), and Merck18 (29, 30). p < 0.05 was defined as statistically significant.

### OncoLnc

OncoLnc (http://www.oncolnc.org/) is a tool that allows the interactive exploration of survival correlations based on the survival data of 8,647 patients from 21 cancer studies conducted in TCGA (31). In this work, the survival analysis for *LAMP2* in ESCA was performed using OncoLnc.

### Analysis of LAMP2-Interacting Genes and Proteins

The *LAMP2* interaction network was constructed using the GeneMANIA database (32) (http://www.genemania.org). The STRING online database (33) (https://string-db.org/) was used to construct the LAMP2 protein–protein interaction network.

### GO and KEGG Pathway Analysis and Gene Set Enrichment Analysis

To predict the function of *LAMP2* and its associated pathways, we performed a correlation analysis between *LAMP2* and other genes in ESCA using TCGA data. GO analysis is an efficient bioinformatics tool for identifying the biological processes, cellular components, and molecular functions linked to the gene of interest. GSEA is a computational method for determining statistical differences in the expression status of a set of genes between two organisms (34). GSEA was used to investigate the potential mechanism of *LAMP2*. We further explored potential functional pathways based on the top 250 genes using the clusterProfiler R package (35) (Version 3.14.3). Adjusted P < 0.05 was considered to indicate the meaningful enrichment of a pathway.

### Tissue Preparation and Immunohistochemistry

Tumor specimens were obtained from 40 consecutive patients undergoing a single surgical resection in the First Affiliated Hospital of Shantou University from 2020 to 2021. None of the patients had received preoperative chemotherapy or radiotherapy. Ethical approval was granted by the clinical research ethics committee of the First Affiliated Hospital of Shantou University. Tumor tissues were fixed in 10% formalin and embedded in paraffin. Paraffin-embedded 4 µm thick tissue sections were automatically immunohistochemically stained using a Ventana BenchMark XT immunostainer (Ventana Medical Systems, Tucson, AZ, United States) with a basic Diaminobenzidine (DAB) Kit (Ventana Cat, Tucson, USA). The specimens were diluted to 1:400 with a prediluted polyclonal anti-lamp2 Rabbit polyclonal antibody (pAb) (Wanleibio, Shenyang, China) and incubated for 24 min. Specimens were restained with hematoxylin.

Immunohistochemical sections were visualized and analyzed after full-slide digitization using the Panoramic Scan and Image Pro-Plus (IPP) software. The density means and integrated optical density (IOD) of IPP are representative parameters for assessing immunostaining quantification, allowing for an increased sensitivity of scoring and enabling a more reliable and reproducible protein expression analysis. To further compare the expression of *LAMP2* in other cancers, the expression of *LAMP2* in five tumor types and the corresponding normal tissues was validated using The Human Protein Atlas (THPA) (https://www.proteinatlas.org/) database (36). Three pairs of samples (cancer and normal tissue) were downloaded for each type of cancer. The density means and IOD of IPP were calculated. The GraphPad Prism software (version 5.0) was used to perform unpaired t-tests (Student’s t-test) on the average IOD values obtained from the cancer and normal tissues. p < 0.05 was defined as statistically significant.

### Statistical Analysis

The R package (version 3.6.3) was used for performing all statistical tests, and the ggplot2 package (3.3.3 version) was used for visualization. Kaplan–Meier survival analyses were performed with the “survival R” and “survminer R” packages in the R software. The ROC analysis was performed with the qROC package (version 1.17.0.1). The immune infiltration algorithm (ssGSVA) in the GSVA package (version 1.34.0) was used to calculate immune scores (37). *LAMP2* differential expression analysis was performed using the DESeq2 package (38) (version 1.26.0). t-test or Wilcoxon rank sum test was used for continuous variables and Pearson’s chi-square test for categorical variables. p < 0.05 was considered to indicate statistical significance (ns, p ≥ 0.05; ^∗^p < 0.05; ^∗∗^p < 0.01; ^∗∗∗^p < 0.001).

## Results

### *LAMP2* Showed Significantly Higher Expression in ESCA and ESCC

**Figure 1:**
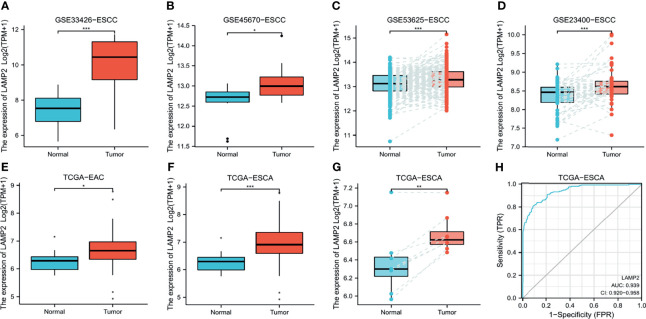
*LAMP2* expression in esophageal cancer. The disparity expression of *LAMP2* based on the GEO database. *LAMP2* mRNA levels in ESCC tumor tissues and normal tissues in the **(A)** GSE33426 (N:12 vs. T:59), **(B)** GSE45670 (N: 10 vs. T: 28), **(C)** GSE53625 (N: 179 vs. T: 179), and **(D)** GSE23400 (N: 53 vs. T: 53) datasets. **(E)** Analysis of *LAMP2* expression in EAC and adjacent normal tissues was examined in the TCGA database (N: 10 vs. T: 80). **(F)** Analysis of LAMP2 expression in TCGA tumors and normal tissues with the data of the GTEx database as controls (N: 666 vs. T: 182). **(G)** TCGA database and statistical analyses of *LAMP2* expression in 8 pairs of ESCA tissues and adjacent normal tissues. **(H)** ROC curve for *LAMP2* expression in TCGA with GTEx (N: 666 vs. T: 182), respectively. TPM, transcript per million; N, normal, T, tumor; *P < 0.05, **P < 0.01, ***P < 0.001.

*LAMP2* expression was identified using the data from different GEO datasets. The expression of *LAMP2* was higher in ESCC tissues than in adjacent normal tissues in the GSE33426 (**Figure 1A**), GSE45670 (**Figure 1B**), GSE53625 (**Figure 1C**), and GSE23400 (**Figure 1D**) datasets. Furthermore, based on TCGA data, LAMP2 protein expression was higher in EAC (**Figure 1E**) and ESCA (**Figures 1F, G**) compared with adjacent normal tissues. The statistical analyses performed on ESCA samples and adjacent normal tissue samples from TCGA showed that *LAMP2* expression had an effective predictive value (area under the curve [AUC] = 0.939) (**Figure 1H**). These findings indicate that *LAMP2* expression is upregulated in ESCA and suggest an important regulatory role of *LAMP2* in ESCA progression.

### Correlations Between *LAMP2 E*xpression and Clinicopathology

**Figure 2:**
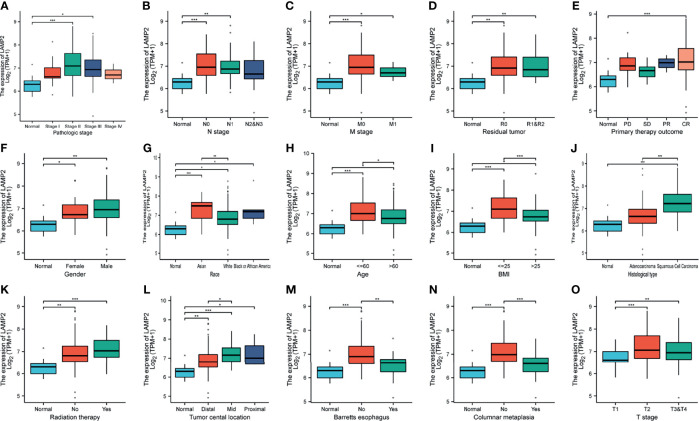
Box plots evaluating *LAMP2* expression among different groups of patients based on clinical parameters using the TCGA database. Analysis is shown for pathologic stage **(A)**, N stage **(B)**, M stage **(C)**, residual tumor **(D)**, primary therapy outcome **(E)**, gender **(F)**, race **(G)**, age **(H)**, BMI **(I)**, histological type **(J)**, radiation therapy **(K)**, tumor central location **(L),** Barrett’s esophagus **(M)**, columnar metaplasis **(N)**, and T stage **(O)**. PD, progressive disease; SD, stable disease; PR, partial response; CR, complete response; BMI, body mass index; TPM, transcript per million; *P < 0.05, **P < 0.01, ***P < 0.001.

We investigated the correlations between *LAMP2* expression and clinicopathological features in ESCA including the pathologic stages, N stage, M stage, residual tumor, primary therapy outcome, gender, race, age, body mass index (BMI), histological type, radiation therapy, tumor central location, Barrett’s esophagus, columnar metaplasia, and T stage. A high expression of *LAMP2* was significantly associated with pathologic stages II and III (**Figure 2A**), N0 and N1 stages (**Figure 2B**), M0 and M1 stages (**Figure 2C**), residual tumor (R0, R1, and R2) (**Figure 2D**), complete response (**Figure 2E**), gender (female, male) (**Figure 2F**), race (Asian, White, Black, or African-American) (**Figure 2G**), age (≤60, >60) (**Figure 2H**), BMI (≤25, >25) (**Figure 2I**), histological type (squamous cell carcinoma) (**Figure 2J**), radiation therapy (**Figure 2K**), tumor central location (distal, mid, proximal) (**Figure 2L**), Barrett’s esophagus (no) (**Figure 2M**), columnar metaplasis (no) (**Figure 2N**) and T2 stage (**Figure 2O**). These correlations of *LAMP2* expression with various clinicopathological features highlight that more attention should be paid to the patients with certain concomitant clinical traits, such as an age over 60 years, the absence of Barrett’s esophagus, or distal central location of the tumor.

### Correlation Analysis Between *LAMP2* Expression and Prognostic Value

**Figure 3:**
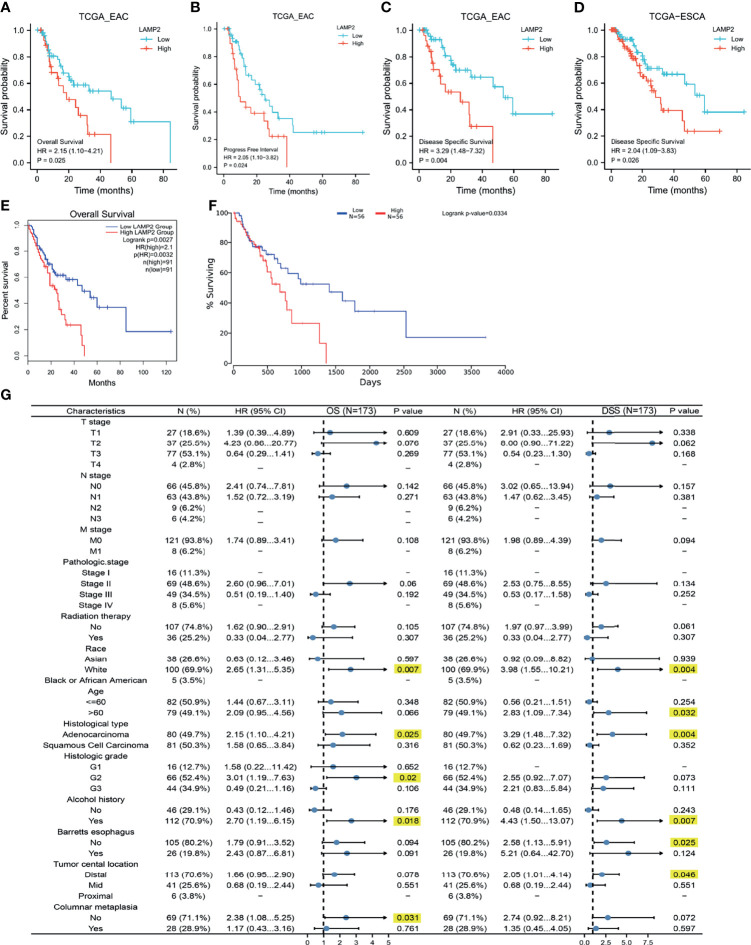
Correlations between *LAMP2* expression and the prognosis (OS, PFI, and DSS) of cancers. **(A–C)** *LAMP2* expression on OS, PFI, and DSS in EAC based on the TCGA database. **(D)** *LAMP2* expression on DSS in ESCA based on the TCGA database. **(E)** *LAMP2* expression on OS in ESCA based on the GEPIA2 database. **(F)** Kaplan plot for *LAMP2* in ESCA based on the OncoLnc. **(G)** Forest plots based on the TCGA database showing *LAMP2* expression and clinicopathological parameters in OS and DSS of ESCA patients. OS, overall survival; PFI, progression-free interval; DSS, disease-specific survival.

Next, we downloaded prognostic information for patients with EAC and ESCA from TCGA to analyze the prognostic value of *LAMP2*. A high expression of *LAMP2* in patients with EAC was significantly associated with worse OS (**Figure 3A**), PFI (**Figure 3B**), and DSS (**Figure 3C**). Subsequently, the prognostic information of patients with ESCA was obtained from TCGA, GEPIA2, and Oncolnc to analyze the prognostic value of *LAMP2.* We investigated the relationship between *LAMP2* expression and prognosis in patients with ESCA. The results showed that a high expression of *LAMP2* was significantly related to worse DSS (**Figure 3D**) in patients with ESCA from TCGA. In the GEPIA2 and Oncolnc data, we found that a high expression of *LAMP2* was associated with worse OS in patients with ESCA (**Figures 3E, F**). These results suggest that LAMP2 is significantly associated with the prognosis of ESCA.

### Validation of the Prognostic Value of *LAMP2* Based on a Variety of Clinicopathological Features

Kaplan–Meier analysis was used to better understand and explore the correlations of *LAMP2* expression with various clinical characteristics in patients with ESCA. As shown in **Figure 3G**, high *LAMP2* expression, adenocarcinoma, and a history of alcohol use were significantly correlated with worse OS and DSS (**Figure 3G**). Moreover, we found that *LAMP2* expression was significantly associated with poor DSS in ESCA patients >60 years of age or without Barrett’s esophagus or a distal tumor central location (**Figure 3G**). The upregulation of *LAMP2* expression was only associated with poorer OS in patients with histological grade 2 and those without columnar metaplasia (**Figure 3G**). These results suggest that *LAMP2* expression has a prognostic value in ESCA.

### Identification of *LAMP2*-Interacting Genes and Proteins

**Figure 4:**
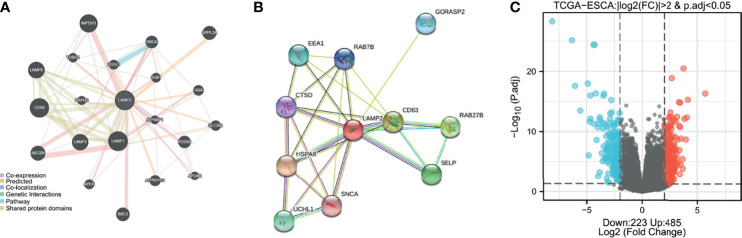
Identification of LAMP2-interacting genes, proteins and DEGs. **(A)** The gene–gene interaction network of *LAMP2* was constructed using GeneMania. **(B)** A visual network of *LAMP2*- binding protein interactions was obtained based on the STRING database. **(C)** Volcano map of differentially expressed genes, with 485 upregulated genes and 223 downregulated genes. Normalized expression levels are shown in descending order from blue to red.

**Figure 5:**
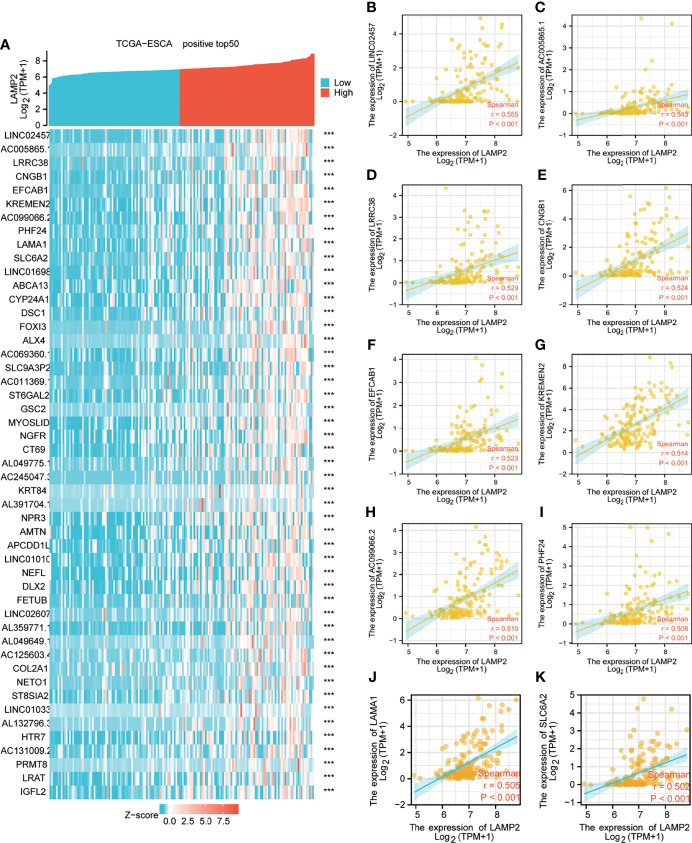
Top 50 genes positively associated with *LAMP2* expression in ESCA. **(A)** The gene coexpression heat map of the top 50 genes positively linked with *LAMP2* in ESCA; correlation analysis of the top 10 genes and *LAMP2* in the heat map: **(B)** *LINC02457*, **(C)** *AC005865.1*, **(D)** *LRRC38* **(E)** *CNGB1*, **(F)** *EFCAB1*, **(G)** *KREMEN2*, **(H)**, *AC099066.2* **(I)** *PHF24*, **(J)** *LAMA1*, and **(K)** *SLC6A2*. ***p < 0.001.

GeneMANIA was used to construct a gene–gene interaction network for *LAMP2*. As shown in **Figure 4A**, the 20 genes closely related to *LAMP2* were identified, including *LAMP1*, *CD60*, *IMPDH1*, *LAMP3*, and *LAMP5* (**Figure 4A**). A protein–protein interaction network for LAMP2 was established using the STRING database (**Figure 5B**). There were 27 edges and 11 nodes, including SLC40A1, TFR2, and HFE (P<0.001) (**Figure 4B**).

### Identification of DEGs in ESCA in *LAMP2* Low-Expression and High-Expression Groups

**Figure 6:**
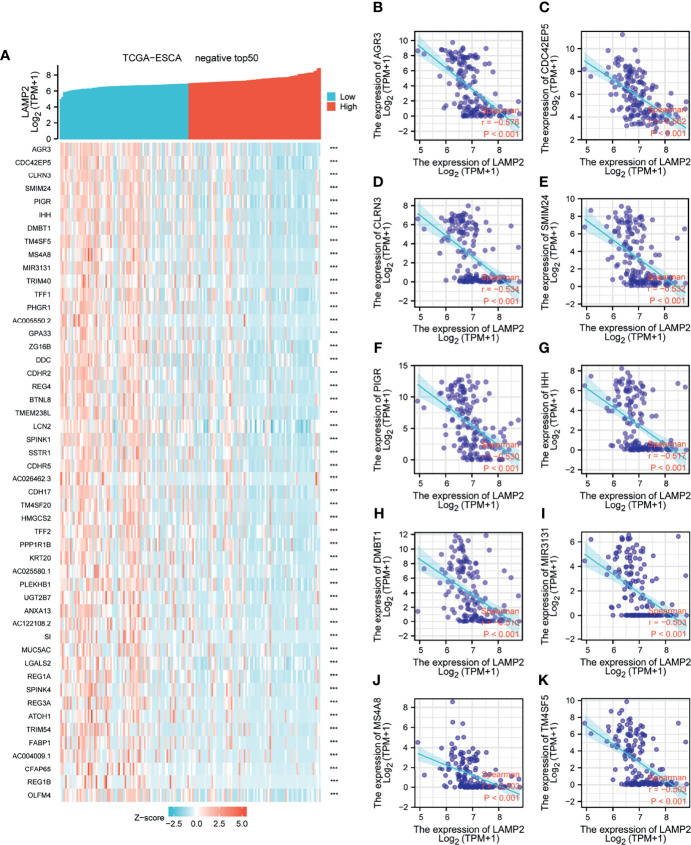
Top 50 genes negatively associated with *LAMP2* expression in ESCA. **(A)** The gene coexpression heat map of the top 50 genes negatively linked with *LAMP2* in ESCA; correlation analysis of the top 10 genes with *LAMP2* in the heat map: **(B)** *AGR3*, **(C)** *CDC42EP5*, **(D)** *CLRN3* **(E)** *SMIM24*, **(F)** *PIGR*, **(G)** *IHH*, **(H)** *DMBT1*, **(I)** *MIR3131*, **(J)** *MS4A8*, and **(K)** *TM4SF5*. ***p < 0.001.

Median *LAMP2* mRNA expression was used to divide samples into high-expression and low- expression groups. A total of 708 DEGs were identified, including 485 upregulated and 223 downregulated genes, between the *LAMP2* low- and high-expression groups (|log fold change| > 2, P < 0.05) (**Figure 4C**). Heat maps were used to illustrate the associations between the top 50 related genes and *LAMP2* expression levels. As shown in the heat map of positive correlations (**Figure 5A**), the expression level of *LAMP2* was positively associated with those of *LINC02457* (r = 0.555) (**Figure 5B**), *AC005865.1* (r = 0.543) (**Figure 5C**), *LRRC38* (r = 0.529) (**Figure 5D**), cyclic nucleotide-gated channel subunit beta 1 (*CNGB1*; r = 0.524) (**Figure 5E**), EFCAB1 (r = 0.523) (**Figure 5F**), *KREMEN2* (r = 0.514) (**Figure 5G**), *AC099066.2* (r = 0.510) (**Figure 5H**), *PHF24* (r = 0.508) (**Figure 5I**), *LAMA1* (r = 0.505) (**Figure 5J**), and *SLC6A2* (r = 0.502) (**Figure 5K**). Among the negatively correlated genes (**Figure 6A**), the top 10 were *AGR3* (r = −0.576) (**Figure 6B**), *CDC42EP5* (r = −0.562) (**Figure 6C**), *CLRN3* (r = −0.534) (**Figure 6D**), *SMIM24* (r = −0.532) (**Figure 6E**), the polymeric immunoglobulin receptor (*PIGR*; r = −0.530) (**Figure 6F**), *IHH* (r = −0.517) (**Figure 6G**), *DMBT1* (r = −0.510) (**Figure 6H**), *MIR3131* (r = −0.503) (**Figure 6I**), *MS4A8* (r = −0.503) (**Figure 6J**), and *TM4SF5* (r = −0.503) (**Figure 6K**).

### Functional Enrichment Analysis

**Figure 7:**
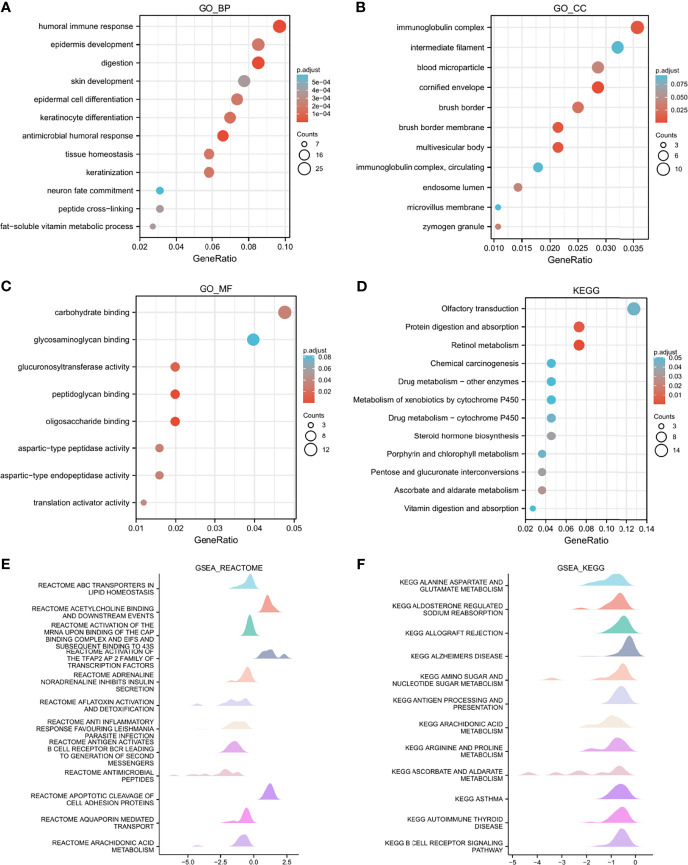
Functional enrichment analysis of LAMP2 in ESCA. **(A–C)** Significant Gene Ontology terms of the top 250 genes of DEGs most positively associated with LAMP2 include BPs, CC, and MF. **(D)** Significant KEGG pathways of the top 250 genes most positively associated with LAMP2. (E, F) Significant GSEA results of the top 250 genes most positively associated with LAMP2, including reactome pathways **(E)** and KEGG pathways **(F)**. FDR<0.25 and p.adjust<0.05. The Gene Ontology and KEGG pathway analysis results for upregulated DEGs with jlog2fold change j >2.

To better comprehend the characteristics of *LAMP2* and its related pathways, we performed a correlation analysis on *LAMP2* and the DEGs in ESCA using TCGA data. The top 250 linked genes most positively correlated with *LAMP2* were picked for enrichment analysis. We further studied the potential functional pathway based on the top 250 genes using the clusterProfiler R package. According to the GO enrichment analysis, the primary biological processes included a humoral immune response, an antimicrobial humoral response, epidermis development, digestion, epidermal cell differentiation, and keratinocyte differentiation. The main cellular components were the immunoglobulin complex, blood microparticle, and cornified envelope. The molecular functions were principally related to carbohydrate binding, glucuronosyltransferase activity, and peptidoglycan binding (**Figures 7A–C**). In addition, KEGG pathway analysis identified the enrichment and crosstalk of the top 250 genes in protein digestion and absorption and retinol metabolism (**Figure 7D**). GSEA was used to search for reactome and KEGG pathways: the ABC transporter in lipid homeostasis; acetylcholine binding and downstream events; activation of the mRNA upon the binding of the cap-binding complex and eukaryotic initiation factors (eIFs), and subsequent binding to 43S; and activation of the TFAP2 (AP-2) family of transcription factors were extremely enriched (**Figure 7E**). Alanine aspartate and glutamate metabolism, aldosterone-regulated sodium reabsorption, allograft rejection, and the allograft rejection pathway were significantly enriched in the KEGG analysis (**Figure 7F**). These results strongly suggest that *LAMP2* is involved in the regulation of the immune response in ESCA.

### Immune Infiltration Analysis in ESCA

**Figure 8:**
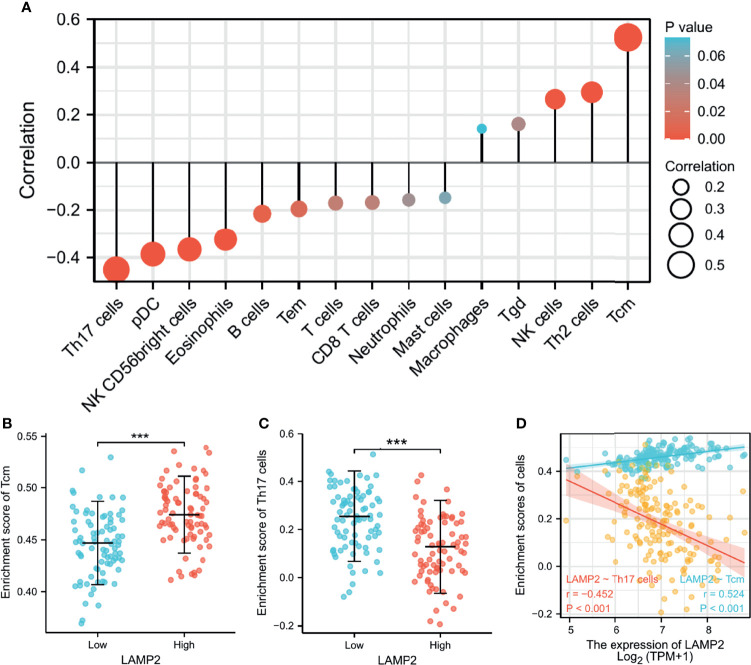
*LAMP2* expression is linked with immune infiltration in the ESCA microenvironment. **(A)** *LAMP2* was positively correlated with 5 immune cells and negatively correlated with 10 immune cells according to the forest plots. **(B, C)** Correlation between the relative enrichment score of Tcm and Th17 cells and the expression level (TPM) of *LAMP2*. **(D)** Relevance between the relative enrichment score of Tcm, Th17 cells, and *LAMP2* expression level. ***p < 0.001.

**Figure 9:**
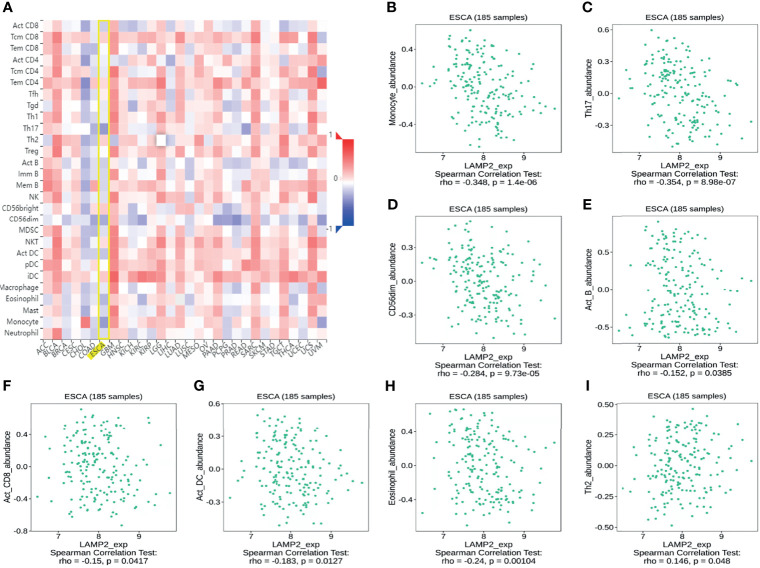
Correlation analysis of *LAMP2* expression with the immune-related signatures of 28 TIL types in cancer based on the TISIDB database. **(A)** The landscape of relationship between *LAMP2* expression and TILs in multiple types of cancers (red indicates positive correlation; blue indicates negative correlation). **(B–H)** *LAMP2* expression was negatively linked with infiltrating levels of Act_DC, CD56dim, eosinophil, Act_B, monocyte, Act_CD8, and Th17 in ESCA. **(I)** *LAMP2* expression was meaningfully positively correlated with infiltrating levels of Th2 in ESCA.

Spearman correlation analysis showed that the expression level of *LAMP2* in ESCA was related to immune cell infiltration levels as quantified by ssGSVA. The size of the dot in **Figure 8A** shows the absolute value of Spearman’s r, indicating that infiltrating cells were correlated with *LAMP2* in the differential expression analysis. Specifically, *LAMP2* was positively correlated with TCM (T central memory) cells, Th2 cells, and natural killer (NK) cells (**Figure 8A**) and negatively correlated with Th17 cells, pDC, NK CD56 bright cells, and eosinophils (**Figure 8A**). The infiltration of TCM and Th17 cells in the *LAMP2* differential expression analysis was statistically significant (P<0.001) (**Figures 8B, C**). The relative enrichment scores of TCM and Th17 cells showed significantly positive and negative correlations with *LAMP2* expression levels, respectively (P<0.001) (**Figure 8D**). Next, we further analyzed the relationship between *LAMP2* expression and the abundance of 28 TILs using TISIDB. **Figure 9A** shows the relationships between *TPM4* expression and *LAMP2* in different types of cancer. In particular, *LAMP2* expression was significantly associated with multiple types of TILs (**Figures 9B–I**) in ESCA. *LAMP2* expression was meaningfully negatively associated with infiltrating levels of monocytes (rho = −0.348, p = 1.4e-06) (**Figure 9B**), Th17 cells (rho = −0.354, p = 8.98e-07) (**Figure 9C**), CD56dim cells (rho = −0.284, p = 9.73e-05) (**Figure 9D**), Act_B cells (rho = −0.152, p = 0.0385) (**Figure 9E**), Act_CD8 cells (rho = −0.15, p = 0.0417) (**Figure 9F**), Act_DC cells (rho = −0.183, p = 0.0127) (**Figure 9G**), and eosinophils (rho = −0.24, p = 0.00104) (**Figure 9H**) and positively correlated with the infiltrating levels of Th2 cells (rho = 0.146, p = 0.048) (**Figure 9I**) in ESCA.

### Pan-Cancer Expression Landscape of *LAMP2*

**Figure 10:**
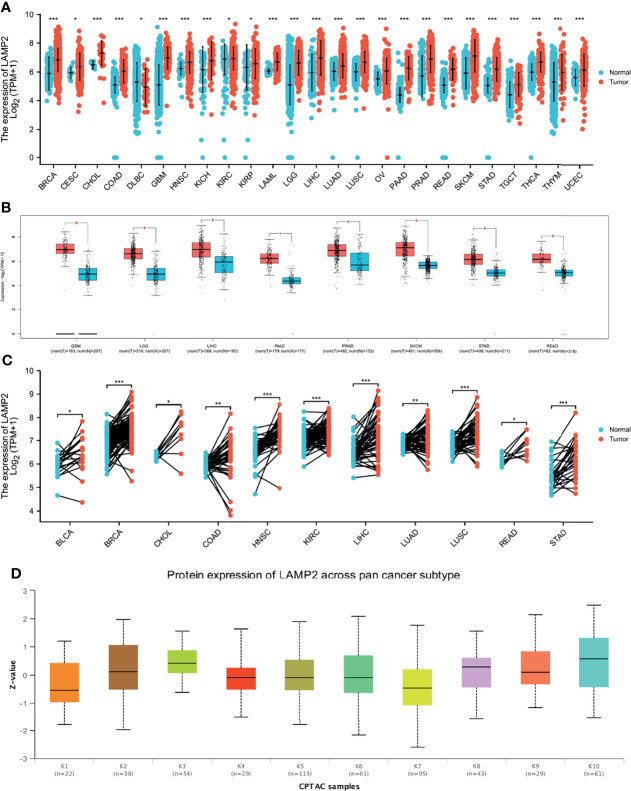
*LAMP2* expression in pan-cancer (ruling out ESCA). **(A)** Expression levels of *LAMP2* in a dataset containing 25 tissues in tumor cell lines, derived from the TCGA data and normal tissues with the data of the GTEx database; **(B)** expression levels of *LAMP2* in a dataset containing 8 tissues in tumor cell lines, derived from the GEPIA2 data; **(C)** *LAMP2* expression in TCGA tumors and adjacent normal tissues; **(D)** Protein expression of LAMP2 across pan-cancer subtype in CPTAC samples based on UALCAN data. (*p < 0.05, **p < 0.01, ***p < 0.001).

The TIMER database was used to analyze *LAMP2* mRNA expression in 25 commonly occurring types of human cancer. *LAMP2* expression was significantly upregulated in 24 cancer types, BRCA, CESC, CHOL, COAD, GBM, HNSC, KICH, KIRC, KIRP, LAML, LGG, LIHC, LUAD, LUSC, OV, PAAD, PRAD, READ, SKCM, STAD, TGCT, THCA, THYM, and UCEC, whereas it was downregulated only in DLBC (**Figure 10A**). Then, *LAMP2* expression levels were determined using the GEPIA2 database. We found that *LAMP2* was significantly more highly expressed in eight human tumor types (GBM, LGG, LIHC, PAAD, PRAD, SKCM, STAD, and READ) compared with normal tumor tissues, a result consistent with those shown in **Figure 10A** (**Figure 10B**). *LAMP2* expression was significantly upregulated in 11 cancer types (BLCA, BRCA, CHOL, COAD, HNSC, KIRC, LIHC, LUAD, LUSC, READ, and STAD (**Figure 10C**). In addition, we analyzed the protein expression of LAMP2 across 10 cancer subtypes in CPTAC samples based on UALCAN data (**Figure 10D**). In CPTAC samples, high *LAMP2* expression was correlated strongly with subtypes k3 and k10. These findings suggest that *LAMP2* may have an important regulatory role in the progression of various cancers.

### Pan-Cancer Diagnostic Value of *LAMP2*

**Figure 11:**
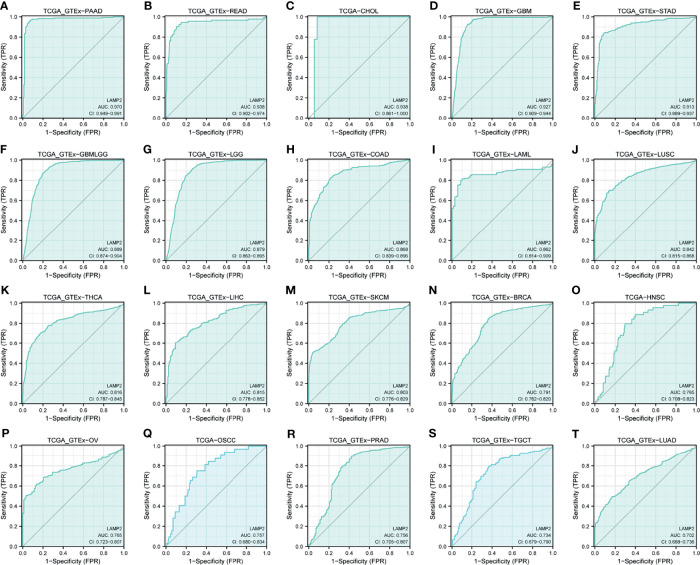
Receiver operating characteristic (ROC) curve for *LAMP2* expression in pan-cancer. **(A)** PAAD; **(B)** READ; **(C)** CHOL; **(D)** GBM; **(E)** STAD; **(F)** GBMLGG; **(G)** LGG; **(H)** COAD; **(I)** LAML; **(J)** LUSC; **(K)** THCA; **(L)** LIHC; **(M)** SKCM; **(N)** BRCA; **(O)** HNSC; **(P)** OV; **(Q)** OSCC; **(R)** PRAD; **(S)** TGCT; and **(T)** LUAD.

ROC curves were constructed to assess the diagnostic value of *LAMP2* in other cancers. The results showed that *LAMP2* had a certain accuracy (AUC > 0.7) in predicting 20 cancer types: PAAD (AUC = 0.970) (**Figure 11A**), READ (AUC = 0.938) (**Figure 11B**), CHOL (AUC = 0.938) (**Figure 11C**), GBM (AUC = 0.927) (**Figure 11D**), STAD (AUC = 0.913) (**Figure 11E**), GBMLGG (AUC = 0.889) (**Figure 11F**), LGG (AUC = 0.879) (**Figure 11G**), COAD (AUC = 0.868) (**Figure 11H**), LAML (AUC = 0.862) (**Figure 11I**), LUSC (AUC = 0.842) (**Figure 11J**), THCA (AUC = 0.816) (**Figure 11K**), LIHC (AUC = 0.815) (**Figure 11L**), SKCM (AUC = 0.803) (**Figure 11M**), BRCA (AUC = 0.791) (**Figure 11N**), HNSC (AUC = 0.765) (**Figure 11O**), OV (AUC = 0.765) (**Figure 11P**), OSCC (AUC = 0.757) (**Figure 11Q**), PRAD (AUC = 0.756) (**Figure 11R**), TGCT (AUC = 0.734) (**Figure 11S**), and LUAD (AUC=0.702) (**Figure 11T**). *LAMP2* had high accuracy in predicting PAAD, READ, CHOL, GBM, and STAD (AUC > 0.9). In addition, *LAMP2* had a better reliability in the remaining cancers (0.7 < AUC < 0.9). These results suggest that *LAMP2* has valid pan-cancer diagnostic value.

### Pan-Cancer Prognostic Value of *LAMP2*

**Figure 12:**
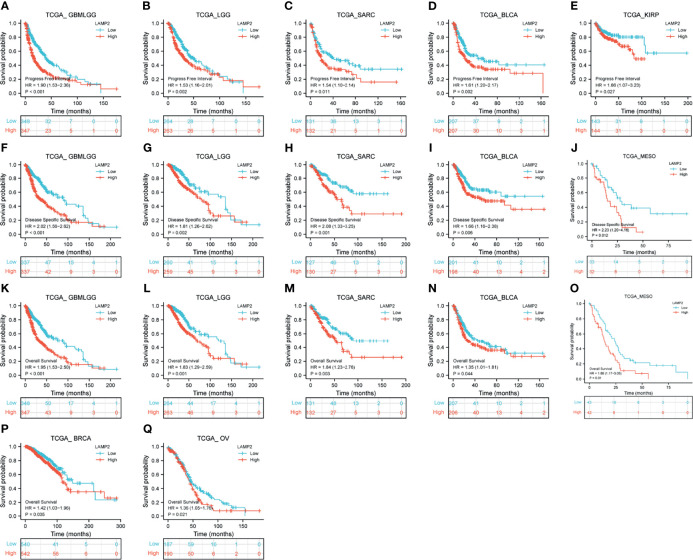
Correlations between *LAMP2* expression and the prognosis (OS, DSS, and PFI) of other cancers. PFI: **(A)** GBMLGG, **(B)** LGG, **(C)** SARC, **(D)** BLCA and **(E)** KIRP; DSS: **(F)** GBMLGG, **(G)** LGG, **(H)** SARC, **(I)** BLCA and **(J)** MESO; and OS: **(K)** GBMLGG, **(L)** LGG, **(M)** SARC, **(N)** BLCA, **(O)** MESO, **(P)** BRCA, and **(Q)** OV.

The expression levels of *LAMP2* were particularly correlated with the PFI, DSS, and OS of patients with GBMLGG, LGG, SARC, and BLCA. Higher *LAMP2* expression was associated with worse PFI, DSS, and OS (P < 0.001) in GBMLGG (**Figures 12A, F, K**); worse PFI (P < 0.01), DSS (P < 0.01) and OS (P < 0.001) in LGG (**Figures 12B, G, L**); worse PFI (P < 0.05), DSS (P < 0.01), and OS (P < 0.01) in SARC (**Figures 12C, H, M**); worse PFI (P < 0.01), DSS (P < 0.01), and OS (P < 0.05) in BLCA (**Figures 12D, I, N**); worse PFI (P < 0.05) in KIRP (**Figure 12E**); worse DSS (P < 0.05) (**Figure 12J**) and OS (P = 0.01) (**Figure 12O**) in MESO; and worse OS (P < 0.05) in BRCA and OV (**Figures 12P, Q**). In addition, we validated the prognostic value of *LAMP2* in a variety of cancers including LUAD, GBMLGG, breast cancer, colorectal cancer, blood cancer (follicular lymphoma), and soft tissue cancer (liposarcoma) using PrognoScan based on the GEO dataset (**Supplementary Table 1**). The results verified the prognostic value of LAMP2 on a pan-cancer basis.

### Correlations Between *LAMP2* and Immune Modulatory Factors Across Multiple Cancer Types

**Figure 13:**
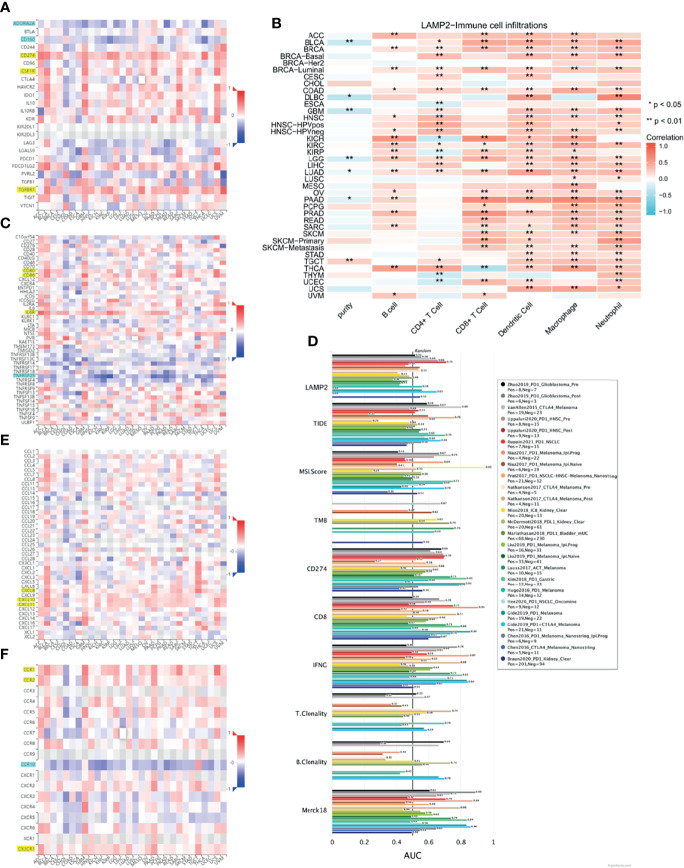
Immune infiltration analysis in pan-cancer. Relationship between *LAMP2* expression and **(A)** immunoinhibitor, **(C)** immunostimulator **(E)** chemokine, and **(F)** receptor in human heterogeneous carcinomas based on the TISIDB database. **(B)** Heat map showing the correlation between infiltration of six immune cells and *LAMP2* expression based on the TIMER2.0 database. **(D)** Bar chart shows the biomarker correlation of *LAMP2* compared to standardized cancer immune evasion biomarkers in the ICB subcohort based on the TIDE algorithm. The AUC was used to evaluate the predictive performance of the tested biomarkers for ICB response status. *p < 0.05, **p < 0.01.

To evaluate the relevance of *LAMP2* to immunity in cancer progression, we explored the relationships between *LAMP2* expression and immunoinhibitors, immunostimulators, chemokines, and receptors in human heterogeneous carcinomas based on TISIDB. As shown in **Figure 13**, *LAMP2* expression levels were positively correlated with various immunoinhibitors (including CD274, the colony-stimulating factor 1 receptor [CSF1R], and TGFBR1) (**Figure 13A**), several immune stimulators (including *CD80*, *CD86*, and *IL6R*) (**Figure 13C**), various chemokines (including *CXCL8*, *CXCL10*, and *CXCL11*) (**Figure 13E**), and multiple receptors (including *CCR1*, *CCR2*, and *CX3CR1*) (**Figure 13F**). In contrast, the immunoinhibitors *ADORA2A* and *CD160*, the immunostimulator *TNFRSF25*, and the receptor *CCR10* were significantly negatively correlated with *LAMP2* expression (**Figures 13A, C, F**). Overall, as shown in **Figure 13B**, there were significant pan-cancer positive correlations between *LAMP2* expression and infiltration of six immune cell types (B cells, CD4+ T cells, CD8+ T cells, macrophages, neutrophils, and dendritic cells). Only six tumor types (BRCA, BRCA-luminal, COAD, KIRC, LGG, and LUAD) were consistently associated with the six immune cell types. The strongest correlations were with B cells in KTCH (r = 0.57, p < 0.01) and THCA (r = 0.52, p < 0.01); with CD4+ T cells in THCA (r = 0.47, p < 0.01) and HNSC-HPVpos (r = 0.44, p < 0.01); with CD8+ T cells in PAAD (r = 0.65, p < 0.01), KICH (r = 0.65, p < 0.01); and PRAD (r = 0.59, p < 0.01); with dendritic cells in PAAD (r = 0.58, p < 0.01) and DLBC (r = 0.55, p < 0.01); with macrophages in PAAD (r = 0.66, p < 0.01) and THCA (r = 0.51, p < 0.01); and with neutrophils in DLBC (r = 0.69, p < 0.01) and SKCM-Primary (r = 0.54, p < 0.01) (all data are from the TIMER2.0 database, and values are given to two decimal places). By contrast, five tumor types (ESCA, KICH, KIRP, THYM, and UCEC) were significantly negatively associated with CD4+ T cells, and only THCA was negatively associated with CD8+ T cells. None of the other cancer types showed any significant correlation (p > 0.05) between *LAMP2* expression levels and tumor infiltration by immune cells (**Figure 13B**).

We assessed the biomarker relatedness of *LAMP2* by comparing it using standardized biomarkers based on predictive power response outcomes and OS in the immune checkpoint blockade (ICB) subcohort. These results showed that *LAMP2* alone had an AUC value >0.5 in 12 of the 25 ICB subcohorts (**Figure 13D**). *LAMP2* showed a higher predictive value than TMB, T. Clonality, and B. Clonality, which respectively had AUC values >0.5 in seven, nine, and seven ICB subcohorts. However, the predictive value of *LAMP2* was lower than those of TIDE, MSI score, CD274, CD8, IFNG, and Merck 18. In summary, *LAMP2* is related to immune modulatory factors in a variety of tumor types.

### *LAMP2* Expression in Immune and Molecular Subtypes of Cancers

**Figure 14:**
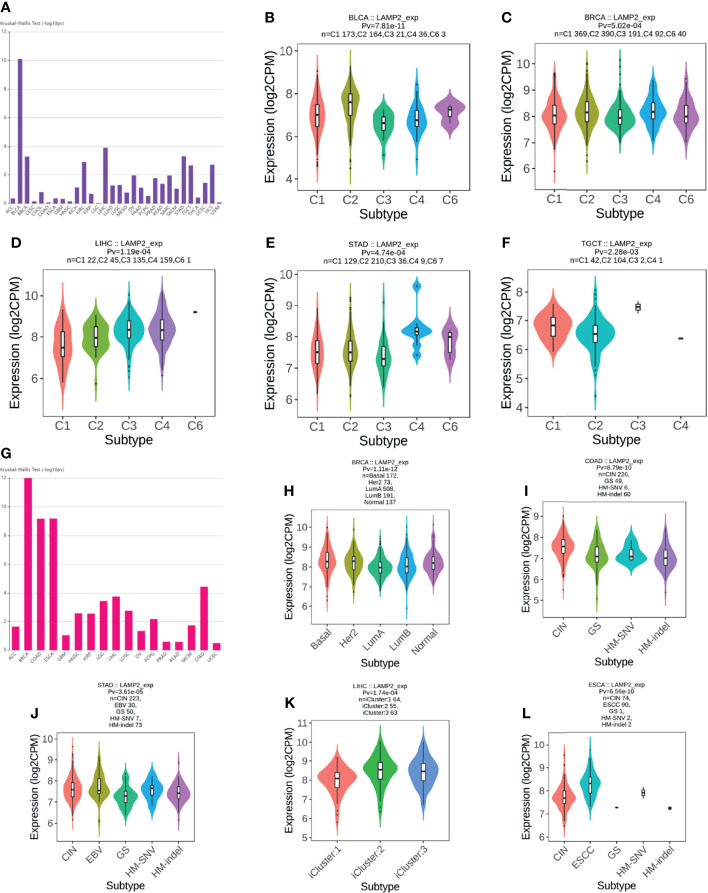
*LAMP2* expression in immune and molecular subtypes of cancers. **(A)** Correlation of *LAMP2* expression with immune subtypes in tumors based on the TISIDB database; **(B)** BLCA; **(C)** BRCA**; (D)** LIHC**; (E)** STAD**;** and **(F)** TGCT**. (G)** Correlation of *LAMP2* expression with molecular subtypes in tumors based on the TISIDB database**; (H)** BRCA**; (I)** COAD**; (J)** STAD**; (K)** LIHC**;** and **(L)** ESCA.

An investigation of the correlations between *LAMP2* expression and tumor molecular subtypes and immune subtypes based on TISIDB showed that *LAMP2* expression was meaningfully linked with several different immune subtypes (C1: wound healing, C2: IFN-gamma dominant, C3: inflammatory, C4: lymphocyte depleted, C5: immunologically quiet, and C6: TGF-b dominant) in five cancer types (**Figure 14A**): BLCA (**Figure 14B**), BRCA (**Figure 14C**), LIHC (**Figure 14D**), STAD (**Figure 14E**), and TGCT (**Figure 14F**). We also found that *LAMP2* was differentially expressed in the diverse molecular subtypes of various tumors (**Figure 14G**), including BRCA, COAD, STAD, LIHC, and ESCA. Specifically, *LAMP2* expression was higher in the Her2 molecular subtype of BRCA (**Figure 14H**), the CIN molecular subtype of COAD (**Figure 14I**), HM-SNV molecular subtype of STAD (**Figure 14J**), iCluster:2 molecular subtype of LIHC (**Figure 14K**), and ESCC subtype of ESCA compared with the other molecular subtypes of the respective tumor types (**Figure 14L**). In conclusion, LAMP2 is highly expressed in the immune and molecular subtypes of some cancer types.

### *LAMP2* Expression Validated by Immunohistochemistry

**Figure 15:**
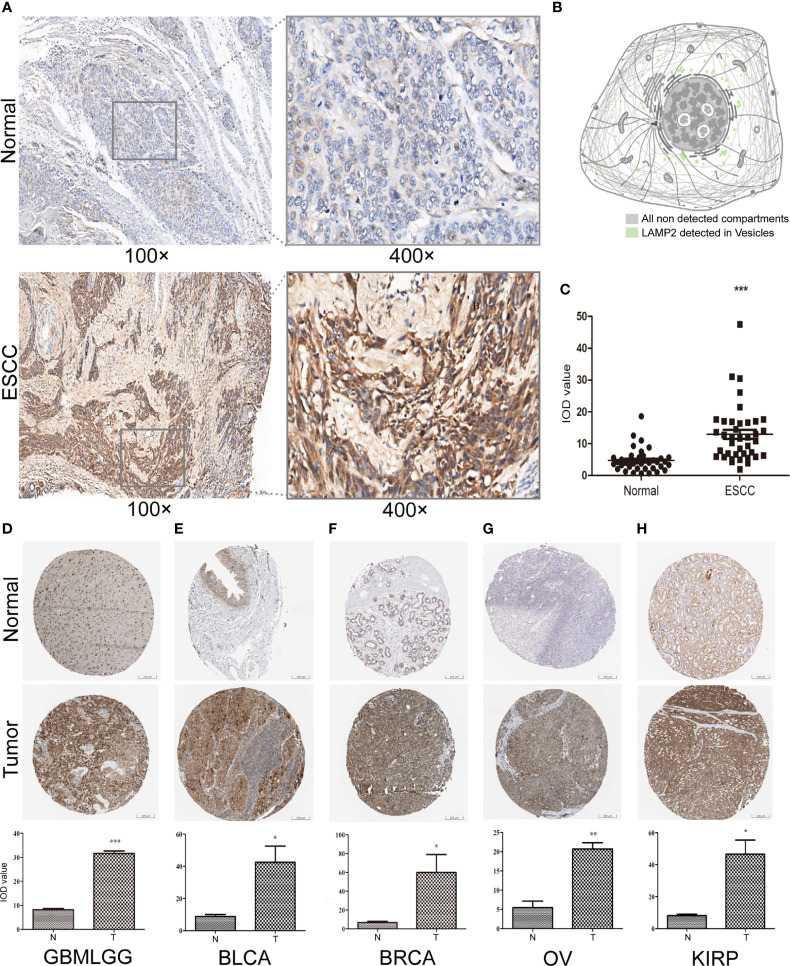
*LAMP2* expression validated by applying immunohistochemistry. **(A)** Expression of *LAMP2* in representative negative and positive specimens of ESCC stained by IHC. **(B)** Subcellular localization of *LAMP2* in cancer cells by the THPA database. Antibody-green. **(C)** Quantitative staining. Black dots indicate the mean density and IOD values of 40 images of ESCC patient tissue and corresponding normal tissue. The expression of *LAMP2* gene was significantly higher in **(D)** GBMLGG, **(E)** BLCA, **(F)** BRCA, **(G)** OV, and **(H)** KIRP expression than in the corresponding normal tissues by the THPA database. *P < 0.05, **P < 0.01, ***P < 0.001.

We performed immunohistochemical staining and found that the expression levels of *LAMP2* protein in ESCC tissues were significantly higher than those in corresponding normal tissues (**Figures 15A, C**). The subcellular localization of *LAMP2* in cancer cells indicated that it was predominantly expressed in cytoplasmic lipid droplets (**Figure 15B**). Finally, the results further confirmed the differences in *LAMP2* expression between the tissues of five tumor types and the corresponding normal tissues from THPA and showed that *LAMP2* was significantly highly expressed in gliomas (**Figure 15D**), BLCA (**Figure 15E**), BRCA (**Figure 15F**), OV (**Figure 15G**), and KIRP (**Figure 15H**).

## Discussion

ESCA is the sixth most common cause of cancer deaths (39). Despite recent advances in molecular marker diagnostics, radiomics, targeted therapies, and immunotherapy, the long-term survival rates of patients with ESCA remain relatively poor (4–6). Therefore, it is of great importance to identify the potential therapeutic targets for ESCA. ESCC and EAC are the main subtypes of ESCA (2, 6). LAMP2, a single transmembrane protein located on the restriction endosomal membranes of lysosomes and advanced nuclear nucleosomes, has an important role in tumor cell metastasis in EAC (24). We found that *LAMP2* was highly expressed with extremely high predictive value (AUC = 0.939) in ESCA; this result was validated by immunohistochemistry. Our findings were consistent with those of previous reports and showed that *LAMP2* could serve as a biomarker to promote the development and progression of ESCA (40). Similarly, studies have confirmed that *LAMP2* can promote cell migration and invasion in some types of cancers, including LUAD (13), LIHC (14), COAD (15), and PRAD (16, 17). However, no previous studies have evaluated the significance of *LAMP2* on a pan-cancer basis. In this study, our findings supplemented those of previous reports to indicate that *LAMP2* has a potential as a new epigenetic biomarker and target that promotes the development and progression of the majority of malignant tumors.

High *LAMP2* expression was related to subtypes k3 and k10 in CPTAC samples (41). Hypoxia affects the tumor microenvironment (TME). Tumor cells secrete a variety of cytokines and chemokines during hypoxia and establish a gradient that creates recruitment or rejection of immune cell subsets in hypoxic areas; this ultimately promotes the formation of an immunosuppressive microenvironment and immune escape and functional angiogenesis, which, in turn, promotes tumor development and metastasis (42). In the k3 subtype, tumor cells may be more likely to develop a hypoxic microenvironment. Hypoxia is known to dysregulate the complement system in various cell types of the TME (41, 43, 44). In the k10 subtype, tumor cells are frequently exposed to endogenous and exogenous factors that alter protein homeostasis, resulting in ER stress. ER stress states have been shown to regulate a variety of precancerous features and the function of dynamically reprogrammed immune cells (45). ER stress may be activated by a variety of factors that interfere with protein folding capacity, resulting in the unfolded protein response and cell death (41, 46). The present study provides some new evidence that a high expression of *LAMP2* in tumor cells may induce tumor growth and metastasis through the formation of a hypoxic microenvironment and ER stress. The exact mechanism requires further experimental investigation.

Our findings also provide an important basis for further comprehensive analysis of the role of *LAMP2* in the clinical subgroups of ESCA. Kaplan–Meier survival analysis suggested that high *LAMP2* expression was significantly correlated with worse prognosis in ESCA. The results were consistent with those of previous research (24). The correlations between *LAMP2* expression and various clinicopathological features highlight the need to pay more attention to patients over the age of 60 and those without Barrett’s esophagus or distal tumor central location in order to improve their clinical outcomes. We found that *LAMP2* was highly expressed in GBMLGG, BLCA, BRCA, OV, and KIRP. These results were validated by immunohistochemistry in TPHA. *LAMP2* has diagnostic and prognostic importance in the above tumor types and is a potential biomarker or therapeutic target for precision treatment of tumors.

GeneMANIA was used to identify 20 genes that were closely related to *LAMP2*, including *LAMP1*, *CD60*, *IMPDH1*, *LAMP3*, and *LAMP5*. *LAMP3* has been reported to be correlated with ESCA (47). Using the STRING database, we also found that SLC40A1, TFR2, and HFE protein expression was meaningfully linked with *LAMP2*. SLC40A1 has been shown to be a significant risk gene with respect to OS in ESCC patients (48). According to functional enrichment analysis, *LAMP2* was mainly associated with immune-related functions, for example, humoral immune response, antimicrobial humoral response, and immunoglobulin complex. Next, heat maps were used to illustrate the top 10 genes positively correlated with *LAMP2*; these were *LINC02457*, *AC005865.1*, *LRRC38*, *CNGB1*, *EFCAB1*, *KREMEN2*, *AC099066.2*, *PHF24*, *LAMA1*, and *SLC6A2*. *CNGB1* is involved in the olfactory pathway and has been found to be overexpressed in ESCC. The expression of *CNGB1* has been proposed as a marker with potential diagnostic or therapeutic value (49). The top 10 genes negatively correlated with *LAMP2* expression were *AGR3*, *CDC42EP5*, *CLRN3*, *SMIM24*, *PIGR*, *IHH*, *DMBT1*, *MIR3131*, *MS4A8*, and *TM4SF5*. PIGR has been considered as a candidate prognostic biomarker in several cancers, and a previous study reported that reduced *PIGR* expression was associated with more aggressive tumors in the distal esophagus (50, 51). The finding that *PIGR* expression was negatively correlated with *LAMP2* further validates the reliability of *LAMP2* as a potentially relevant biomarker.

Accelerated tumor progression is not only associated with malignant cells but is also influenced by the TME (52). As researchers continue to understand and appreciate the tumor immune microenvironment, there is a great potential to develop the ability to predict and guide the responses to immunotherapy (53). Other studies have shown that the TME promotes tumor proliferation and metastasis by producing growth factors, chemokines, and matrix-degrading enzymes and supporting tumor cells (54). Our results show that *LAMP2* expression in ESCA and other tumors is positively correlated with tumor-infiltrating immune cells. For example, *LAMP2* gene expression levels were positively correlated with those of immunoinhibitors *CD274* (PD-L1) and *CSF1R* in a variety of cancers. Different types of cancers show high expression levels of PD-L1 and use PD-L1/PD-1 signaling to evade T-cell immunity (55). The use of CSF1R inhibitors in cancer therapy is currently of great interest, with various therapeutic approaches targeting its ligand or receptor in clinical development (56). Through functional enrichment analysis, we found that the mechanism of *LAMP2* in ESCA may also be primarily associated with the aforementioned immune-related genes. We also found the protein expression of LAMP2 across a pan-cancer subtype; high *LAMP2* protein expression was associated with the k3 subtype of the innate immune system. These findings suggest a possible impact of *LAMP2* expression on the tumor immune microenvironment. Further elucidating the interactions between tumors and immune cells will help to predict immunotherapeutic responses and develop new immunotherapeutic targets. In addition, we showed that *LAMP2* was more strongly associated with certain immune and molecular subtypes in multiple cancer types. Notably, in BRCA, STAD, and LIHC, *LAMP2* was strongly associated with both molecular and immune subtypes. Hence, the studies focusing on a unique molecular subtype or immune subtype may help determine the potential mechanism of action of *LAMP2* and demonstrate that *LAMP2* is a promising diagnostic pan-cancer biomarker that is involved in immune regulation. Ultimately, *LAMP2*-related studies and new targeted therapies may help to improve the poor prognosis of patients with esophageal and other cancers.

There were some limitations to this study. First, we explored *LAMP2* using GEO, TCGA, GTExase, and other public databases, but there was a lack of actual clinical data. Second, we used immunohistochemistry to validate the results in ESCC tissues but lacked EAC specimens. The data from immunohistochemistry experiments on other tumor types were obtained from public databases. Further precise validation by biological experiments is required.

In summary, we found that *LAMP2* may have a role in predicting poor prognosis and relate to the level of immune infiltration in ESCA and other cancers. Thus, *LAMP2* could serve as a novel prognostic biomarker and provide an opportunity and challenge to develop new immunotherapy strategies.

## Data Availability Statement

The original contributions presented in the study are included in the article/**Supplementary Material**. Further inquiries can be directed to the corresponding authors.

## Ethics Statement

The studies involving human participants were reviewed and approved by the clinical research ethics committee of the First Affiliated Hospital of Shantou University. The patients/participants provided their written informed consent to participate in this study. Written informed consent was not obtained from the individual(s) for the publication of any potentially identifiable images or data included in this article.

## Author Contributions

ZX and SL conceptualized and designed the study. SX and SZ collected data. SL and YL provided the tissue specimen and staining. XL and DL performed the data analysis. ZX and SL designed and revised the manuscript. All authors contributed to the article and approved the submitted version.

## Funding

This work was supported by the National Natural Science Foundation of China (No.81001340), the Medical Scientific Research Foundation of Guangdong Province, China (A2021474), and the Medical and Health Science and Technology Project of Shantou, Guangdong, China (210526156491330).

## Conflict of Interest

The authors declare that the research was conducted in the absence of any commercial or financial relationships that could be construed as a potential conflict of interest.

## Publisher’s Note

All claims expressed in this article are solely those of the authors and do not necessarily represent those of their affiliated organizations, or those of the publisher, the editors and the reviewers. Any product that may be evaluated in this article, or claim that may be made by its manufacturer, is not guaranteed or endorsed by the publisher.

## References

[1] Smyth EC Lagergren J Fitzgerald RC Lordick F Cunningham D. Oesophageal Cancer. Nat Rev Dis Primers (2017) 3:17048. doi: 10.1038/nrdp.2017.48 28748917 PMC6168059

[2] Talukdar FR di Pietro M Secrier M Moehler M Goepfert K Lima SSC. Molecular Landscape of Esophageal Cancer: Implications for Early Detection and Personalized Therapy. Ann NY Acad Sci (2018) 1434(1):342–59. doi: 10.1111/nyas.13876 29917250

[3] Yang H Hu B. Recent Advances in Early Esophageal Cancer: Diagnosis and Treatment Based on Endoscopy. Postgrad Med (2021) 133(6):665–73. doi: 10.1080/00325481.2021.1934495 34030580

[4] Yang YM Hong P Xu WW He QY Li B. Advances in Targeted Therapy for Esophageal Cancer. Signal Transduct Target Ther (2020) 5(1):229. doi: 10.1038/s41392-020-00323-3 33028804 PMC7542465

[5] Sah BR Owczarczyk K Siddique M Cook GJR Goh V. Radiomics in Esophageal and Gastric Cancer. Abdom Radiol (New York) (2019) 44(6):2048–58. doi: 10.1007/s00261-018-1724-8 PMC6934409 30116873

[6] Liu K Zhao T Wang J Chen Y Zhang R Lan X. Etiology, Cancer Stem Cells and Potential Diagnostic Biomarkers for Esophageal Cancer. Cancer Lett (2019) 458:21–8. doi: 10.1016/j.canlet.2019.05.018 PMC6597177 31125642

[7] Alessandrini F Pezzè L Ciribilli Y. LAMPs: Shedding Light on Cancer Biology. Semin Oncol (2017) 44(4):239–53. doi: 10.1053/j.seminoncol.2017.10.013 29526252

[8] Ballabio A Bonifacino JS. Lysosomes as Dynamic Regulators of Cell and Organismal Homeostasis. Nat Rev Mol Cell Biol (2020) 21(2):101–18. doi: 10.1038/s41580-019-0185-4 31768005

[9] Tang T Yang ZY Wang D Yang XY Wang J Li L. The Role of Lysosomes in Cancer Development and Progression. Cell Biosci (2020) 10(1):131. doi: 10.1186/s13578-020-00489-x 33292489 PMC7677787

[10] Loeffler DA Klaver AC Coffey MP Aasly JO. Cerebrospinal Fluid Concentration of Key Autophagy Protein Lamp2 Changes Little During Normal Aging. Front Aging Neurosci (2018) 10:130. doi: 10.3389/fnagi.2018.00130 29867441 PMC5952035

[11] Damaghi M Tafreshi NK Lloyd MC Sprung R Estrella V Wojtkowiak JW. Chronic Acidosis in the Tumour Microenvironment Selects for Overexpression of LAMP2 in the Plasma Membrane. Nat Commun (2015) 6:8752. doi: 10.1038/ncomms9752 26658462 PMC4682176

[12] Tan KP Ho MY Cho HC Yu J Hung JT Yu AL. Fucosylation of LAMP-1 and LAMP-2 by FUT1 Correlates With Lysosomal Positioning and Autophagic Flux of Breast Cancer Cells. Cell Death Dis (2016) 7(8):e2347. doi: 10.1038/cddis.2016.243 27560716 PMC5108328

[13] Li QK Shah P Li Y Aiyetan PO Chen J Yung R. Glycoproteomic Analysis of Bronchoalveolar Lavage (BAL) Fluid Identifies Tumor-Associated Glycoproteins From Lung Adenocarcinoma. J Proteome Res (2013) 12(8):3689–96. doi: 10.1021/pr400274w PMC4238415 23802180

[14] Huang PS Lin YH Chi HC Tseng YH Chen CY Lin TK. Dysregulated FAM215A Stimulates LAMP2 Expression to Confer Drug-Resistant and Malignant in Human Liver Cancer. Cells (2020) 9(4):13–7. doi: 10.3390/cells9040961 PMC7227021 32295144

[15] Bednarczyk M Fatyga E Dzięgielewska-Gęsiak S Waniczek D Grabarek B Zmarzły N. The Expression Patterns of BECN1, LAMP2, and PINK1 Genes in Colorectal Cancer Are Potentially Regulated by Micrornas and CpG Islands: An In Silico Study. J Clin Med (2020) 9(12):13–17. doi: 10.3390/jcm9124020 PMC7764710 33322704

[16] Jamali L Moradi A Ganji M Ayati M Kazeminezhad B Fazeli Attar Z. Potential Prognostic Role for SPOP, DAXX, RARRES1, and LAMP2 as an Autophagy Related Genes in Prostate Cancer. Urol J (2020) 17(2):156–63. doi: 10.22037/uj.v0i0.4935 30882175

[17] Morell C Bort A Vara-Ciruelos D Ramos-Torres Á Altamirano-Dimas M Díaz-Laviada I. Up-Regulated Expression of LAMP2 and Autophagy Activity During Neuroendocrine Differentiation of Prostate Cancer LNCaP Cells. PloS One (2016) 11(9):e0162977. doi: 10.1371/journal.pone.0162977 27627761 PMC5023108

[18] Tomczak K Czerwińska P Wiznerowicz M. The Cancer Genome Atlas (TCGA): An Immeasurable Source of Knowledge. Contemp Oncol (Poznan Poland) (2015) 19(1a):A68–77. doi: 10.5114/wo.2014.47136 PMC4322527 25691825

[19] Barrett T Wilhite SE Ledoux P Evangelista C Kim IF Tomashevsky M. NCBI GEO: Archive for Functional Genomics Data Sets–Update. Nucleic Acids Res (2013) 41(Database issue):D991–5 doi: 10.1093/nar/gks1193 PMC3531084 23193258

[20] Su H Hu N Yang HH Wang C Takikita M Wang QH. Global Gene Expression Profiling and Validation in Esophageal Squamous Cell Carcinoma and Its Association With Clinical Phenotypes. Clin Cancer Res (2011) 17(9):2955–66. doi: 10.1158/1078-0432.CCR-10-2724 PMC3086948 21385931

[21] Yan W Shih J Rodriguez-Canales J Tangrea MA Player A Diao L. Three-Dimensional mRNA Measurements Reveal Minimal Regional Heterogeneity in Esophageal Squamous Cell Carcinoma. Am J Pathol (2013) 182(2):529–39. doi: 10.1016/j.ajpath.2012.10.028 PMC3562732 23219752

[22] Li J Chen Z Tian L Zhou C He MY Gao Y. LncRNA Profile Study Reveals a three-lncRNA Signature Associated With the Survival of Patients With Oesophageal Squamous Cell Carcinoma. Gut (2014) 63(11):1700–10. doi: 10.1136/gutjnl-2013-305806 PMC4215280 24522499

[23] Wen J Yang H Liu MZ Luo KJ Liu H Hu Y. Gene Expression Analysis of Pretreatment Biopsies Predicts the Pathological Response of Esophageal Squamous Cell Carcinomas to Neo-Chemoradiotherapy. Ann Oncol (2014) 25(9):1769–74. doi: 10.1093/annonc/mdu201 24907633

[24] Wang Y Liang N Xue Z Xue X. Identifying an Eight-Gene Signature to Optimize Overall Survival Prediction of Esophageal Adenocarcinoma Using Bioinformatics Analysis of ceRNA Network. Onco Targets Ther (2020) 13:13041–54. doi: 10.2147/OTT.S287084 PMC7764560 33376353

[25] Tang Z Kang B Li C Chen T Zhang Z. GEPIA2: An Enhanced Web Server for Large-Scale Expression Profiling and Interactive Analysis. Nucleic Acids Res (2019) 47(W1):W556–60. doi: 10.1093/nar/gkz430 PMC6602440 31114875

[26] Chandrashekar DS Bashel B Balasubramanya SAH Creighton CJ Ponce-Rodriguez I Chakravarthi B. UALCAN: A Portal for Facilitating Tumor Subgroup Gene Expression and Survival Analyses. Neoplasia (New York NY) (2017) 19(8):649–58. doi: 10.1016/j.neo.2017.05.002 PMC5516091 28732212

[27] Mizuno H Kitada K Nakai K Sarai A. PrognoScan: A New Database for Meta-Analysis of the Prognostic Value of Genes. BMC Med Genomics (2009) 2:18. doi: 10.1186/1755-8794-2-18 19393097 PMC2689870

[28] Ru B Wong CN Tong Y Zhong JY Zhong SSW Wu WC. TISIDB: An Integrated Repository Portal for Tumor-Immune System Interactions. Bioinformatics (Oxford England) (2019) 35(20):4200–2. doi: 10.1093/bioinformatics/btz210 30903160

[29] Jiang P Gu S Pan D Fu J Sahu A Hu X. Signatures of T Cell Dysfunction and Exclusion Predict Cancer Immunotherapy Response. Nat Med (2018) 24(10):1550–8. doi: 10.1038/s41591-018-0136-1 PMC6487502 30127393

[30] Fu J Li K Zhang W Wan C Zhang J Jiang P. Large-Scale Public Data Reuse to Model Immunotherapy Response and Resistance. Genome Med (2020) 12(1):21. doi: 10.1186/s13073-020-0721-z 32102694 PMC7045518

[31] Anaya J. OncoLnc: Linking TCGA Survival Data to mRNAs, miRNAs, and lncRNAs. PeerJ Computer Science (2016) 2(2). doi: 10.7717/peerj-cs.67

[32] Franz M Rodriguez H Lopes C Zuberi K Montojo J Bader GD. GeneMANIA Update 2018. Nucleic Acids Res (2018) 46(W1):W60–w4. doi: 10.1093/nar/gky311 PMC6030815 29912392

[33] Szklarczyk D Gable AL Nastou KC Lyon D Kirsch R Pyysalo S. The STRING Database in 2021: Customizable Protein-Protein Networks, and Functional Characterization of User-Uploaded Gene/Measurement Sets. Nucleic Acids Res (2021) 49(D1):D605–12. doi: 10.1093/nar/gkaa1074 PMC7779004 33237311

[34] Subramanian A Tamayo P Mootha VK Mukherjee S Ebert BL Gillette MA. Gene Set Enrichment Analysis: A Knowledge-Based Approach for Interpreting Genome-Wide Expression Profiles. Proc Natl Acad Sci USA (2005) 102(43):15545–50. doi: 10.1073/pnas.0506580102 PMC1239896 16199517

[35] Yu G Wang LG Han Y He QY. Clusterprofiler: An R Package for Comparing Biological Themes Among Gene Clusters. Omics (2012) 16(5):284–7. doi: 10.1089/omi.2011.0118 PMC3339379 22455463

[36] Colwill K Gräslund S. A Roadmap to Generate Renewable Protein Binders to the Human Proteome. Nat Methods (2011) 8(7):551–8. doi: 10.1038/nmeth.1607 21572409

[37] Hänzelmann S Castelo R Guinney J. GSVA: Gene Set Variation Analysis for Microarray and RNA-Seq Data. BMC Bioinform (2013) 14:7. doi: 10.1186/1471-2105-14-7 PMC3618321 23323831

[38] Love MI Huber W Anders S. Moderated Estimation of Fold Change and Dispersion for RNA-Seq Data With Deseq2. Genome Biol (2014) 15(12):550. doi: 10.1186/s13059-014-0550-8 25516281 PMC4302049

[39] Secrier M Li X de Silva N Eldridge MD Contino G Bornschein J. Corrigendum: Mutational Signatures in Esophageal Adenocarcinoma Define Etiologically Distinct Subgroups With Therapeutic Relevance. Nat Genet (2017) 49(2):317. doi: 10.1038/ng0217-317a 28138154

[40] Zhang L Gao Y Zhang X Guo M Yang J Cui H. TSTA3 Facilitates Esophageal Squamous Cell Carcinoma Progression Through Regulating Fucosylation of LAMP2 and ERBB2. Theranostics (2020) 10(24):11339–58. doi: 10.7150/thno.48225 PMC7532669 33042286

[41] Chen F Chandrashekar DS Varambally S Creighton CJ. Pan-Cancer Molecular Subtypes Revealed by Mass-Spectrometry-Based Proteomic Characterization of More Than 500 Human Cancers. Nat Commun (2019) 10(1):5679. doi: 10.1038/s41467-019-13528-0 31831737 PMC6908580

[42] Riera-Domingo C Audigé A Granja S Cheng WC Ho PC Baltazar F. Immunity, Hypoxia, and Metabolism-The Ménage À Trois of Cancer: Implications for Immunotherapy. Physiol Rev (2020) 100(1):1–102. doi: 10.1152/physrev.00018.2019 31414610

[43] Olcina MM Balanis NG Kim RK Aksoy BA Kodysh J Thompson MJ. Mutations in an Innate Immunity Pathway Are Associated With Poor Overall Survival Outcomes and Hypoxic Signaling in Cancer. Cell Rep (2018) 25(13):3721–32.e6. doi: 10.1016/j.celrep.2018.11.093 30590044 PMC6405289

[44] Olcina MM Kim RK Melemenidis S Graves EE Giaccia AJ. The Tumour Microenvironment Links Complement System Dysregulation and Hypoxic Signalling. Br J Radiol (2019) 92(1093):20180069. doi: 10.1259/bjr.20180069 29544344 PMC6435069

[45] Chen X Cubillos-Ruiz JR. Endoplasmic Reticulum Stress Signals in the Tumour and Its Microenvironment. Nat Rev Cancer (2021) 21(2):71–88. doi: 10.1038/s41568-020-00312-2 33214692 PMC7927882

[46] Cubillos-Ruiz JR Bettigole SE Glimcher LH. Tumorigenic and Immunosuppressive Effects of Endoplasmic Reticulum Stress in Cancer. Cell (2017) 168(4):692–706. doi: 10.1016/j.cell.2016.12.004 28187289 PMC5333759

[47] Yang C Shen S Zheng X Ye K Sun Y Lu Y. Long Noncoding RNA HAGLR Acts as a microRNA-143-5p Sponge to Regulate Epithelial-Mesenchymal Transition and Metastatic Potential in Esophageal Cancer by Regulating LAMP3. FASEB J (2019) 33(9):10490–504. doi: 10.1096/fj.201802543RR 31311326

[48] Zhang RY Liu ZK Wei D Yong YL Lin P Li H. UBE2S Interacting With TRIM28 in the Nucleus Accelerates Cell Cycle by Ubiquitination of P27 to Promote Hepatocellular Carcinoma Development. Signal Transduct Target Ther (2021) 6(1):64. doi: 10.1038/s41392-020-00432-z 33589597 PMC7884418

[49] Ma F Laster K Nie W Liu F Kim DJ Lee MH. Heterogeneity Analysis of Esophageal Squamous Cell Carcinoma in Cell Lines, Tumor Tissues and Patient-Derived Xenografts. J Cancer (2021) 12(13):3930–44. doi: 10.7150/jca.52286 PMC8176252 34093800

[50] Fristedt R Gaber A Hedner C Nodin B Uhlén M Eberhard J. Expression and Prognostic Significance of the Polymeric Immunoglobulin Receptor in Esophageal and Gastric Adenocarcinoma. J Trans Med (2014) 12:83. doi: 10.1186/1479-5876-12-83 PMC4021601 24694107

[51] Gologan A Acquafondata M Dhir R Sepulveda AR. Polymeric Immunoglobulin Receptor-Negative Tumors Represent a More Aggressive Type of Adenocarcinomas of Distal Esophagus and Gastroesophageal Junction. Arch Pathol Lab Med (2008) 132(8):1295–301. doi: 10.5858/2008-132-1295-PIRTRA 18684029

[52] Giraldo NA Sanchez-Salas R Peske JD Vano Y Becht E Petitprez F. The Clinical Role of the TME in Solid Cancer. Br J Cancer (2019) 120(1):45–53. doi: 10.1038/s41416-018-0327-z 30413828 PMC6325164

[53] Binnewies M Roberts EW Kersten K Chan V Fearon DF Merad M. Understanding the Tumor Immune Microenvironment (TIME) for Effective Therapy. Nat Med (2018) 24(5):541–50. doi: 10.1038/s41591-018-0014-x PMC5998822 29686425

[54] Belli C Trapani D Viale G D'Amico P Duso BA Della Vigna P. Targeting the Microenvironment in Solid Tumors. Cancer Treat Rev (2018) 65:22–32. doi: 10.1016/j.ctrv.2018.02.004 29502037

[55] Cha JH Chan LC Li CW Hsu JL Hung MC. Mechanisms Controlling PD-L1 Expression in Cancer. Mol Cell (2019) 76(3):359–70. doi: 10.1016/j.molcel.2019.09.030 PMC6981282 31668929

[56] Cannarile MA Weisser M Jacob W Jegg AM Ries CH Rüttinger D. Colony-Stimulating Factor 1 Receptor (CSF1R) Inhibitors in Cancer Therapy. J Immunother Cancer (2017) 5(1):53. doi: 10.1186/s40425-017-0257-y 28716061 PMC5514481

