# Supplementary material for: LAMP2 as a Biomarker Related to Prognosis and Immune Infiltration in Esophageal Cancer and Other Cancers: A Comprehensive Pan-Cancer Analysis
Source: Front Oncol. 2022 Apr 21;12:884448. doi: 10.3389/fonc.2022.884448 (PMC9069144; doi:10.3389/fonc.2022.884448)
Supplement: Supplementary file 1 [file Table_1.docx]

Supplementary Material

# Supplementary Tables

**Supplementary Table1: Correlations between *LAMP2* expression and the prognosis (OS, RFS, DSS, DMFS, DFS and DRFS) of several cancers** **based on GEO data by the** **PrognoScan data.** OS: Overall Survival; RFS: Relapse Free Survival; DSS: Disease Specific Survival; DMFS: Distant Metastasis Free Survival; DRFS: Distant Recurrence Free Survival.

| **Cancer-type** | **Dataset** | **Probe ID** | **Subtype** | **N** | **Endpoint** | **Cox *P*-value** | **HR [95% CI]** |
| --- | --- | --- | --- | --- | --- | --- | --- |
| Lung cancer | GSE31210 | 203041_s_at | Adenocarcinoma | 204 | OS | 0.000016 | 6.68 [2.82 - 15.83] |
|  | GSE31210 | 203042_at | Adenocarcinoma | 204 | OS | 0.000151 | 5.36 [2.25 - 12.79] |
|  | GSE31210 | 200821_at | Adenocarcinoma | 204 | OS | 0.025266 | 4.80 [1.21 - 19.00] |
|  | GSE31210 | 203041_s_at | Adenocarcinoma | 204 | RFS | 0.000005 | 4.04 [2.22 - 7.36] |
|  | GSE31210 | 203042_at | Adenocarcinoma | 204 | RFS | 0.001607 | 2.77 [1.47 - 5.22] |
|  | GSE31210 | 203042_at | Adenocarcinoma | 204 | RFS | 0.039728 | 2.39 [1.04 - 5.50] |
|  | GSE31210 | 226671_at | Adenocarcinoma | 204 | RFS | 0.030314 | 2.01 [1.07 - 3.79] |
|  | GSE1321 | A_23_P416608 | Adenocarcinoma | 117 | OS | 0.021669 | 1.56 [1.07 - 2.27] |
| Brain cancer | GSE4412-GPL96 | 203041_s_at | Glioma | 74 | OS | 0.001558 | 3.63 [1.63 - 8.05] |
|  | GSE4412-GPL96 | 203042_at | Glioma | 74 | OS | 0.001079 | 3.42 [1.64 - 7.15] |
|  | GSE4412-GPL96 | 200821_at | Glioma | 74 | OS | 0.006196 | 2.05 [1.23 - 3.43] |
|  | MGH-glioma | 38403_at | Glioma | 50 | OS | 0.001551 | 1.94 [1.29 - 2.92] |
| Breast cancer | GSE1456-GPL96 | 203041_s_at |  | 159 | DSS | 0.020158 | 3.17 [1.20 - 8.39] |
|  | GSE11121 | 203042_at |  | 200 | OS | 0.046075 | 2.29 [1.01 - 5.17] |
|  | GSE12093 | 203042_at |  | 200 | DMFS | 0.046075 | 2.29 [1.01 - 5.17] |
|  | GSE2034 | 200821_at |  | 286 | DMFS | 0.001329 | 1.94 [1.30 - 2.92] |
|  | GSE12276 | 203042_at |  | 204 | RFS | 0.003689 | 1.72 [1.19 - 2.47] |
|  | GSE9893 | 5457 |  | 155 | OS | 0.000034 | 1.51 [1.24 - 1.84] |
|  | GSE12276 | 200821_at |  | 204 | RFS | 0.031521 | 1.49 [1.04 - 2.14] |
|  | GSE12276 | 203041_s_at |  | 204 | RFS | 0.037096 | 1.39 [1.02 - 1.89] |
|  | GSE17537 | 203041_s_at |  | 55 | DFS | 0.042572 | 2.71 [1.03 - 7.12] |
|  | GSE17537 | 200821_at |  | 55 | DFS | 0.029062 | 2.59 [1.10 - 6.08] |
|  | GSE14333 | 203041_s_at |  | 226 | DFS | 0.040017 | 1.78 [1.03 - 3.10] |
| Blood cancer | GSE8970 | 200821_at | Follicular lymphoma | 34 | OS | 0.014088 | 2.27 [1.18 - 4.35] |
| Soft tissue cancer | GSE30929 | 203042_at | Liposarcoma | 140 | DRFS | 0.03037 | 1.40 [0.67 - 2.91] |
